# Progressive Asymptomatic Hyperglycemia and Glucosuria Identified Through Protocol-Driven Monitoring in a Clinical Trial: A Case Report

**DOI:** 10.7759/cureus.108555

**Published:** 2026-05-09

**Authors:** Raymond T Anasobi, Omar Stewart, Ronald Stumbris

**Affiliations:** 1 Internal Medicine, Healthcare Research Network, Tinley Park, USA; 2 Internal Medicine, All Saints University School of Medicine, Roseau, DMA; 3 Clinical Research, Healthcare Research Network, Tinley Park, USA

**Keywords:** asymptomatic hyperglycemia, clinical trial monitoring, glycosuria, insulin therapy, medication non adherence, social determinants of health, type 2 diabetes mellitus

## Abstract

Severe hyperglycemia may remain clinically silent despite significant metabolic derangement, particularly in patients with inconsistent engagement in care. We report the case of a 50-year-old patient with type 2 diabetes mellitus enrolled in a gout clinical trial, in which protocol-driven laboratory monitoring revealed progressive, asymptomatic hyperglycemia and worsening glucosuria across multiple study visits. Fasting blood glucose increased from 250 mg/dL at baseline to 474 mg/dL at Week 24, while urine glucose exceeded 1000 mg/dL from Week 16 through Week 24. Hemoglobin A1c was 13%, and the patient had class II obesity with a body mass index of 39.7 kg/m². Despite these findings, the patient denied classic symptoms of hyperglycemia and demonstrated no evidence of acute metabolic decompensation. Evaluation of investigational product safety data did not support a drug-related cause. Further assessment identified medication nonadherence, inconsistent primary care follow-up, and socioeconomic barriers as likely contributors. Following coordination with the patient’s primary care physician, insulin therapy was initiated, resulting in rapid improvement in fasting blood glucose and complete resolution of glucosuria, although fasting glucose remained elevated at Week 32. This case demonstrates that protocol-driven monitoring can uncover clinically silent metabolic deterioration and highlights the critical role of adherence and social determinants of health in diabetes outcomes. Early identification and timely intervention remain essential to preventing progression to life-threatening complications.

## Introduction

Diabetes mellitus is a chronic metabolic disorder associated with substantial morbidity and mortality when glycemic control is not achieved. Persistent hyperglycemia drives microvascular and macrovascular complications, including nephropathy, neuropathy, retinopathy, and cardiovascular disease [1]. While symptomatic hyperglycemia often prompts evaluation, severe hyperglycemia may remain clinically silent, particularly in patients with inconsistent engagement in care [2,3].

Social determinants of health, including financial instability, medication inaccessibility, and limited healthcare utilization, are key contributors to poor glycemic control [4,5]. These factors can delay recognition of disease progression, limit treatment escalation, and increase the risk of preventable complications [3].

This case demonstrates progressive, asymptomatic worsening of hyperglycemia identified through structured, protocol-driven laboratory monitoring within a clinical trial setting. Serial assessment of glycemic and metabolic parameters enabled the detection of clinically significant deterioration that may have been missed in routine care. Preserved renal function and stable metabolic indices despite marked hyperglycemia provide an important clinical context, and evaluation of investigational product safety data did not support the investigational product as a likely contributor, underscoring the role of nonpharmacologic factors in disease progression.

## Case presentation

A 50-year-old male patient with type 2 diabetes mellitus diagnosed in 2015 was enrolled in a clinical trial for gout and underwent routine laboratory monitoring per study protocol, during which progressively worsening hyperglycemia was identified over time. Prior outpatient diabetes management consisted of metformin 500 mg twice daily, and the patient had no prior insulin use before re-engagement with primary care during the study period. The patient had a body weight of 151.1 kg and a body mass index of 39.7 kg/m², consistent with class II obesity and significant insulin resistance.

**Figure 1:**
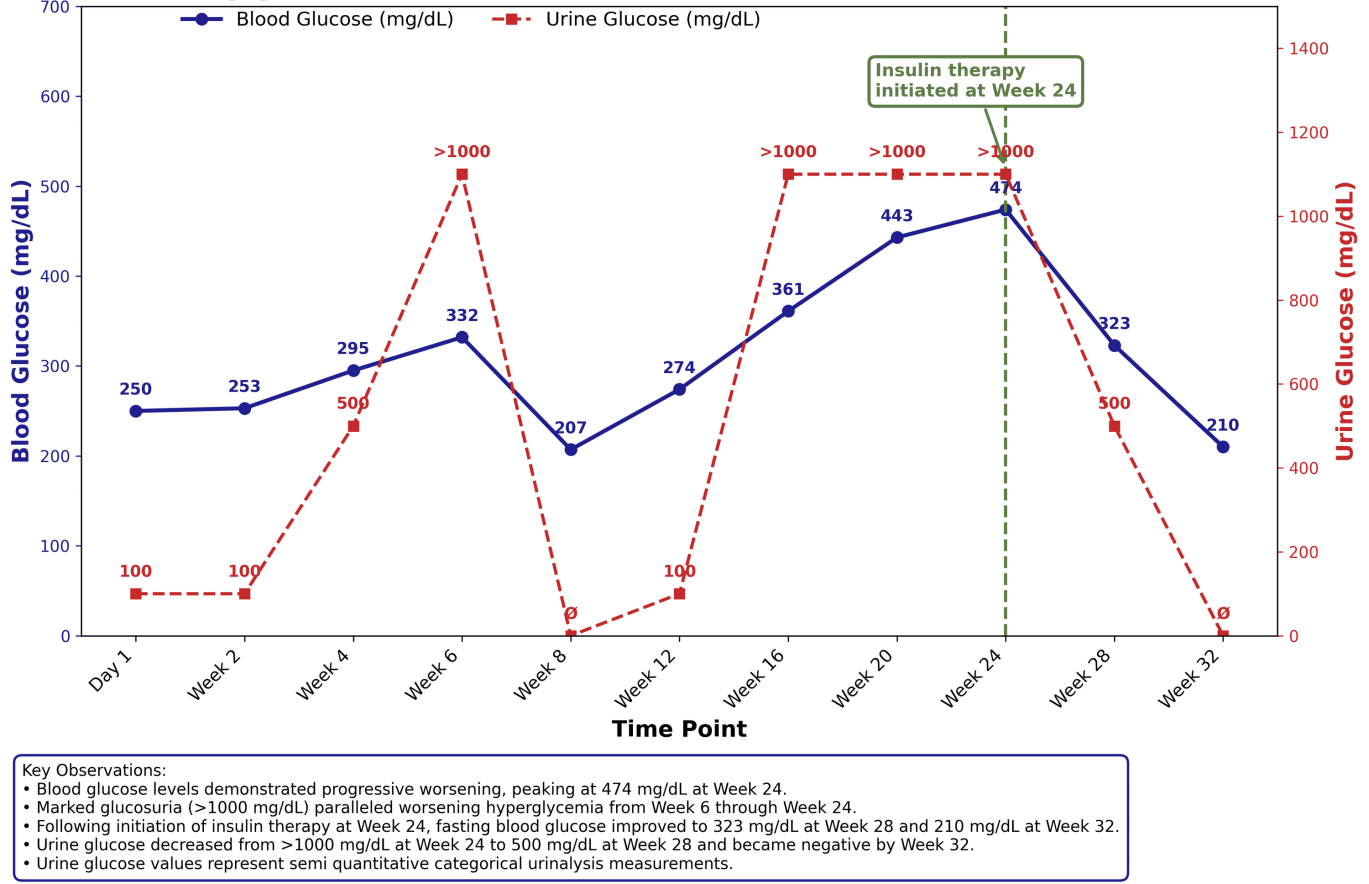
Serial blood glucose and urine glucose trends over time

All laboratory values reported were obtained in the fasting state. Serial fasting blood glucose measurements demonstrated a gradual upward trend, increasing from 250 mg/dL at baseline to a peak of 474 mg/dL at Week 24, followed by improvement to 210 mg/dL by Week 32 after initiation of insulin therapy. These findings are summarized in Figure 1.

Additional metabolic and renal laboratory parameters remained stable throughout monitoring. Estimated glomerular filtration rate (eGFR) ranged from 91 to 106 mL/minute/1.73 m², creatinine from 0.85 to 1.01 mg/dL, sodium from 137 to 139 mmol/L, blood urea nitrogen (BUN) from 8 to 14 mg/dL, and bicarbonate from 23 to 28 mmol/L over time (Table 1). These findings supported preserved renal function and absence of clinically significant metabolic acidosis or dehydration despite progressive hyperglycemia. Serum osmolality was not directly measured; however, available laboratory and clinical findings did not support a hyperosmolar hyperglycemic state. Serum ketones, venous blood gas analysis, and measured serum osmolality were not obtained because the patient remained clinically stable without symptoms or laboratory evidence suggestive of diabetic ketoacidosis or hyperosmolar hyperglycemic state. HbA1c was 13%, consistent with chronic, poorly controlled diabetes mellitus.

**Table 1:** Laboratory results of patient over time eGFR: estimated glomerular filtration rate; Na: sodium; BUN: blood urea nitrogen; HCO3: bicarbonate

Time Point	Blood Glucose (mg/dL)	Urine Glucose (mg/dL)	eGFR (mL/min/1.73m^2^)	Na (*mmol/L)*	BUN (mg/dL)	HCO3 (mmol/L)	Creatinine (mg/dL)
Day 1	250	100	104	137	14	23	0.89
Week 2	253	100	91	139	14	25	1.01
Week 4	295	500	102	139	11	23	0.92
Week 6	332	>1000	105	139	12	25	0.87
Week 8	207	Negative	102	139	12	23	0.92
Week 12	274	100	99	139	11	23	0.93
Week 16	361	>1000	98	138	8	25	0.94
Week 20	443	>1000	99	138	12	28	0.94
Week 24	474	>1000	104	137	13	23	0.89
Week 28	323	500	106	138	12	26	0.85
Week 32	210	Negative	103	139	14	23	0.91

Despite progressively worsening glucose levels, the patient denied classic symptoms of hyperglycemia, including polyuria, polydipsia, fatigue, or altered mental status. Vital signs were stable. Laboratory evaluation demonstrated no evidence of acute metabolic decompensation, with stable vital signs, preserved renal function, and no laboratory evidence of clinically significant metabolic acidosis. Urine glucose measurements were assessed semi-quantitatively and converted to approximate categorical equivalents for consistency in longitudinal presentation.

Given the patient’s participation in a gout clinical trial, a comprehensive evaluation of the investigational product was performed. The investigational product was a selective urate transporter 1 inhibitor under evaluation for gout and hyperuricemia management. Review of available safety information, temporal association, and the Investigator’s Brochure did not identify hyperglycemia as a known or expected adverse effect associated with this drug class. Based on this assessment, the investigational product was not considered a likely contributor to the worsening hyperglycemia.

Further assessment revealed poor adherence to prescribed metformin therapy and inconsistent follow-up with the primary care provider. The patient reported socioeconomic barriers, including inconsistent access to medications and healthcare services, which were identified as likely contributors to disease progression.

The clinical research team facilitated re-engagement with the patient’s primary care physician, and a coordinated management plan was implemented. Insulin therapy was initiated with insulin lispro 15 units before meals and insulin glargine 50 units once daily, with dosing determined and titrated by the primary care physician based on clinical judgment, severity of hyperglycemia, and insulin resistance.

Following initiation of insulin therapy, subsequent study measurements demonstrated improvement in glycemic control, with fasting blood glucose values decreasing to 323 mg/dL at Week 28 and 210 mg/dL at Week 32. Although substantial improvement was observed following insulin initiation, fasting glucose levels at Week 32 remained above recommended glycemic targets, indicating the need for continued outpatient diabetes management and longitudinal follow-up. Resolution of glucosuria despite persistent fasting hyperglycemia at Week 32 may reflect variability in renal glucose threshold, hydration status, timing of urine sampling, and limitations of semi-quantitative urine glucose testing.

## Discussion

This case highlights several clinically important aspects of diabetes management, particularly the potential for severe hyperglycemia to remain asymptomatic despite significant metabolic derangement. The patient in this report exhibited a progressive rise in fasting blood glucose accompanied by marked glucosuria, yet denied classic symptoms of hyperglycemia. No evidence of diabetic ketoacidosis or hyperosmolar hyperglycemic state was identified during the monitoring period. This underscores the limitations of symptom-based assessment and reinforces the importance of objective laboratory monitoring, especially in patients with inconsistent engagement in care [1,4,5].

The temporal relationship between rising glucose levels and worsening glucosuria further illustrates the physiologic threshold at which renal glucose excretion becomes pronounced. Sustained glucosuria exceeding 1000 mg/dL from Week 16 through Week 24 reflects prolonged exposure to significant hyperglycemia, increasing the risk of both microvascular and macrovascular complications if left unaddressed, as well as progression to acute metabolic emergencies such as hyperosmolar hyperglycemic state [6].

Importantly, this case also highlights the critical role of social determinants of health in glycemic control. Medication nonadherence, limited healthcare access, and inconsistent follow-up were identified as key contributors to disease progression, consistent with prior literature demonstrating the significant impact of adherence barriers on glycemic outcomes [3,6]. 

The marked improvement in both blood glucose and glucosuria following initiation of insulin therapy at Week 24 reinforces the effectiveness of timely intervention. By week 32, glycemic control improved rapidly, with a parallel resolution of glucosuria. This response not only confirms the reversibility of severe metabolic derangements with appropriate treatment but also emphasizes the importance of early recognition and intervention [7].

Finally, this case demonstrates the unique value of structured laboratory monitoring within clinical trial settings. Protocol-driven assessments enabled early identification of clinically silent deterioration that may have otherwise gone undetected in routine care. This highlights an opportunity to integrate more systematic and proactive strategies into standard clinical practice, particularly for high-risk populations [8].

The degree of hyperglycemia and elevated HbA1c in this case exceeded recommended glycemic targets and placed the patient at increased risk for acute metabolic complications, including hyperosmolar hyperglycemic state [2,9]. Medication nonadherence was identified clinically as a likely contributor to poor glycemic control [10]. The broader influence of social determinants on diabetes outcomes further supports the need for comprehensive, patient-centered approaches to management [11].

This report describes a single patient and is therefore limited in generalizability. Urine glucose was assessed semi-quantitatively, which may limit precise correlation with serum glucose levels. Additionally, medication adherence and socioeconomic barriers were assessed clinically without the use of validated adherence or standardized social determinants screening tools. Future studies evaluating structured laboratory monitoring in high-risk diabetic populations may help determine its role in routine clinical practice. 

## Conclusions

This case demonstrates that severe, progressive hyperglycemia can remain clinically silent despite significant metabolic deterioration, emphasizing the limitations of symptom-based assessment in diabetes management. Protocol-driven laboratory monitoring enabled early detection of worsening glycemic control and marked glucosuria, which were identified as likely associated with medication nonadherence and socioeconomic barriers, while investigational product effects were not considered likely contributors based on available safety assessments.

Timely initiation of insulin therapy resulted in rapid improvement in fasting blood glucose and complete resolution of glucosuria, although fasting glucose remained above recommended targets at Week 32, underscoring the reversibility of metabolic derangements with appropriate intervention. These findings highlight the importance of proactive monitoring strategies and addressing social determinants of health to improve outcomes in high-risk patients.
